# A multi-modal dataset for learning-based morphology-informed building performance modeling

**DOI:** 10.1038/s41597-026-07886-w

**Published:** 2026-07-21

**Authors:** Sarah Mokhtar, Caitlin Mueller

**Affiliations:** Massachusetts Institute of Technology, Cambridge, USA

## Abstract

Building performance, including airflow and radiative exchanges, is governed by morphology. High-fidelity simulations are accurate but computationally expensive, limiting their use in design workflows. Data-driven approaches provide a scalable alternative, yet their development remains constrained by the absence of high-quality datasets that pair geometrically expressive 3D models with validated physical simulation outputs. Existing datasets are predominantly derived from footprint extrusions, photogrammetric reconstruction, or LoD1-2 city models, and are limited in their suitability for physics-based simulation workflows. They frequently omit critical morphological features, lack watertightness and topological consistency, and do not include associated simulation fields. As a result, performance-informed, shape-driven research often relies on single-use, non-public datasets tailored to narrowly scoped tasks, severely limiting broader adoption, scalability, and integration into data-driven design workflows. This work introduces PRISM (Performance Representation Informed by Shape and Morphology), a publicly available dataset that systematically links building geometry with high-fidelity simulation outputs across multiple environmental domains. PRISM features high morphological diversity, spanning basic extrusions to highly articulated forms; multi-modal geometric representations, including watertight surface meshes, signed distance fields and structured descriptors; and cross-domain environmental and physical simulation outputs from computational fluid dynamics (velocity, pressure) and visibility-derived solar exposure (sky view factor by sky patch). PRISM supports a wide range of research applications, including surrogate modeling, geometry-conditioned performance prediction, inverse design, generative modeling, and large-scale design-space exploration.

## Background & Summary

The influence of building form on wind flow, solar exposure, thermal comfort and structural loading is governed by nonlinear physical processes that are highly sensitive to geometric articulation. Climate and resource constraints motivate early-stage integration of environmental performance into design, when form has the greatest impact. While high-fidelity simulations are often required to capture these effects accurately, their use is constrained by computational overhead, geometric preprocessing requirements, and poor suitability for rapid iteration. Recent advances in data-driven modeling have enabled scalable alternatives to high-fidelity simulation, spanning methods that operate on parametric or descriptor-based geometric inputs^1–5^, structured grid or image representations^6–15^ and unstructured or field-based representations^16,17^. Applications have spanned predominantly urban wind and pedestrian comfort prediction^1,5,7–10,13– 15,17^, with additional directions addressing pollution dispersion and air quality estimation^2,16^, microclimate and thermal comfort modeling^3,12^ and solar radiation approximation^6,11^. These approaches, built on physically grounded data, can produce fast and differentiable estimates of key performance metrics, supporting real-time feedback during iterative design and optimization. Beyond computational acceleration, they support geometry-conditioned performance landscape exploration^18–20^, sensitivity and morphology-performance trade-off analysis^21^ and performance-informed generative design workflows^22,23^. These capabilities can be integrated with qualitative and spatial considerations central to architectural design. By capturing patterns of formal variation, data-driven models can complement physics-based predictors, supporting creativity-aware generation and the synthesis of alternatives that balance performance with spatial and aesthetic intent. The efficacy of such methods, however, hinges on datasets that are both morphologically diverse and simulation-aligned, linking high-resolution 3D geometry with simulated physical fields across multiple environmental modalities. Despite the proliferation of publicly available geospatial and morphological datasets, most fall short of the requirements for simulation-based learning. Common sources include footprint-extruded datasets (e.g., OpenStreetMap^24^, Microsoft Building Footprints^25^), remote sensing reconstructions^26,27^, and authoritative 3D city models (e.g., Boston^28^, Netherlands^29^, Zurich^30^) conforming to LoD1–2 standards^31^. These datasets, while broadly useful, were not designed for physics-based modeling^32^. They often lack key geometric features known to influence environmental behavior, such as podium massing, lift-up conditions, or porosity, and are typically non-watertight or topologically inconsistent, requiring extensive preprocessing for use in numerical solvers^33–35^. Critically, they are not simulation-aligned: they lack associated physical fields and metadata needed to reproduce or evaluate performance. As a result, prior research frequently relies on bespoke parametric datasets, which are often unpublished, narrowly scoped, and under-documented, limiting reproducibility, generalization, and cross-study comparability. This absence of standardized, performance-paired geometry datasets remains a major bottleneck for scalable, data-driven modeling in the built environment.

To address this gap, **PRISM (Performance Representation Informed by Shape and Morphology)** is introduced as a publicly available dataset that systematically links geometry with physical quantities across multiple geometric and environmental data modalities. PRISM is characterized by three key attributes:

- **High morphological diversity**: A broad range of synthetic building geometries, spanning simple extrusions to articulated forms that reflect design-relevant and performance-sensitive geometric variations.
- **Multi-modal geometric representation**: Explicit representations including watertight meshes and boundary representations, implicit volumetric fields (signed distance functions), point clouds, and structured descriptors.
- **Cross-domain environmental and physical simulation outputs**: Validated outputs across multiple domains, including CFD (velocity, pressure) and solar and sky exposure (SVF by sky patch).

PRISM fills a longstanding gap by providing a geometry-to-performance dataset that combines building morphological expressivity, geometric modality and cross-domain physical outputs in a unified, simulation-aligned and reproducible framework. It offers a distinct contribution to performance-informed building design, supporting research in surrogate modeling, geometry-aware performance inference, sensitivity and trade-off analysis and performance-driven design generation. While *building performance* encompasses a wide range of domains including energy, daylighting, acoustics among others, many of these depend not only on geometry but also on material, operational, climatic and interior spatial conditions. The input modalities presented in this dataset are therefore intended both as direct performance signals and as geometry-aware inputs for downstream coupled and proxy analyses, with the focus of *PRISM* centered specifically on capturing the influence of morphology and spatial articulation on environmental and physical performance phenomena.

### Related Works

The advancement of data-driven modeling across domains such as scientific computing, fluid dynamics, engineering design, computer vision, and geometric learning has been driven by the emergence of standardized open-source datasets that couple high-resolution inputs with structured task-aligned outputs. In physics-based domains, simulation-aligned datasets such as AirfRANS^36^, DrivAerNet++^37^, ShapeNet-Car^38^, and AircraftVerse^39^ support supervised learning for complex flow fields, geometry-conditioned surrogate modeling, and inverse design workflows. They provide consistent simulation parameters, mesh-based representations, and physical performance metrics (e.g., drag, lift, pressure fields), enabling reproducible model development and evaluation across a variety of aerodynamic configurations. Similarly, in computer vision and 3D shape analysis, datasets like ImageNet^40^, ShapeNet^41^, and ModelNet^42^ have played a foundational role in advancing classification, part segmentation, generative modeling, and representation learning. Despite analogous modeling challenges in the built environment, where physical behavior is tightly regulated by geometric form, there exists no public dataset that integrates expressive 3D building geometry with validated simulation outputs across modalities such as wind flow and solar exposure. As a result, research in building performance modeling continues to rely on fragmented, proprietary data generated for specific pipelines, limiting reproducibility, scalability, and the broader utility of data-driven methods in architectural contexts.

Table 1 summarizes key limitations in existing datasets relevant to performance-informed building design. Because publicly available geometry-to-performance datasets are virtually non-existent, a range of datasets reported across prior research are included, even when they are not accessible, to provide a comprehensive overview.

**Table 1:** Comprehensive comparison of geometry-to-performance datasets for building and urban applications. The table highlights differences in geometry representations, shape complexity, design parameterization, and performance outputs. Most existing datasets focus on basic or multi-extrusion geometries with low design variability, limited building-level complexity and few performance modalities, primarily velocity. Radiation and comfort metrics are rarely included, and publicly available datasets remain extremely scarce. The proposed PRISM dataset addresses these limitations with broader geometric modalities, higher variability, and more comprehensive performance outputs.

Dataset	Size	Geometry Modalities	Shape Complexity	Type	Design Param.	Variability		Scale	Perf. Modalities		Spatial Coverage	Open- source
						B	U		Aerodyn.	Microcl.		
He *et al*.^1^	300	M	BM	P	3	L	—	B	U	—	G	No
Kakav. *et al*.^16^	50	M, SDF	BM	P	8	L	L	MB	U	—	G, F	No
Lee *et al*.^2^	72	M	BM	P	3	L	M	Bl	U, D	—	G	No
Musil *et al*.^44^	3500	M, V	BM	P	4	L	M	Bl	U	—	G, F, S	No
Tanaka *et al*.^45^	3600*	M	BM	P	9	L	M	Bl	U	—	G, F	No
Wu *et al*.^3^	400	M, P	BM	P	10	L	M	N	U		G, C	No
Zhao *et al*.^4^	200	M, P	ME	M	3	L	—	N	U	T	G	No
Han *et al*.^6^	8992	M, V	ME	P	7	M	L	MB	—	Rad	S	No
Low *et al*.^7^	4352*	M, HM	ME	G	—	M	M	N	U	—	G	No
Tan *et al*.^8^	1600	M	ME	P	un.	M	M	Bl	U	—	G	No
Shao *et al*.^17^	500	M	ME	G	—	M	M	N	U	—	G, F	No
Mokhtar *et al*.^9^	1025	M, HM	ME	P	10	M	H	N	U	—	G, F	No
Galanos *et al*.^11^	25860	M, HM	ME	G	—	M	H	N	—	Rad	G	No
Huang *et al*.^12^	2665	M	ME	P	13	M	H	Bl	U	Rad, UTCI	G	No
Hoiness *et al*.^13^	2384	M, SDF, HM	ME	M	6	M	H	N	U	—	G, S	No
Kastner *et al*.^14^	564	M, SDF, HM	ME	M	4	M	H	N	U	—	G	No
Chen *et al*.^46^	369	M	ME	G	—	M	H	N	—	Rad	S	No
Weeras. *et al*.^5^	150	M	**DM**	P	8	L	—	B	U	—	G, F	No
Clarke *et al*.^15^	3920*	M, HM	**DM**	P	10	L	L	MB	U	—	G, F, S	No
Kim *et al*.^47^	38	M	**DM**	M	7	M	—	N	U	—	G, F	No
Zhang *et al*.^48^	15	M	**DM**	P	1	M	—	B	U	—	G	No
Mokhtar *et al*.^10^	288*	M, HM	**DM**	G	—	M	H	N	U	—	G, F	No
Tanaka *et al*.^49^	28	M	**DM**	P	12	**H**	—	B	F, P	—	S	No
Nazarian *et al*.^43^	538	HM, P	ME	M	4	M	H	N	U, P		G, F, C	**Yes**
PRISM (ours)	5500	BR, M, PC, P	**DM**	P	19	**H**	—	B	U, P	SVF_byP, *Rad*	G, S, F	**Yes**

**Abbreviations**. *Field dim*. = Field dimensionality (2D, 2.5D, 3D). *Cont*. = Field continuity (D = Discrete, C = Continuous). *Pred*. = Predicted field coverage (S = Surface, F = Field, FS = Field slice, PF = Partial field). *Const*. = Geometry constraint (R = Resolution, T = Topology). *Depend*. = Domain dependency (F = Fixed, PF = Partially fixed / shared grid, – = Domain-agnostic). *Mem. scaling* = Computational memory complexity with number of samples *N*. *Geom. opt*. = Geometry-aware optimization capacity (N = None, ID = Indirect / template-based deformation, D = Direct / learned implicit embedding).

Key limitations observed in the current dataset landscape include:

- **Lack of publicly accessible datasets enabling reproducibility and scalability**: To date, UrbanTales^43^ is the only known dataset in the built environment domain that provides high-resolution wind simulation outputs. While it constitutes a substantial contribution to urban climatology, it is designed for neighborhood-scale analysis, relies on extruded 2.5D massing, and does not resolve building-scale morphological variation.
- **Limited shape complexity and intra-dataset morphological variability**: Most datasets focus on basic^1– 3,16,44,45^ or multi-height extrusion^4,6–9,11–14,17,43,46^ models and fail to capture detailed shape features such as lift-up conditions, porosity, articulation, or façade undercuts. As shown in Table 1, the few datasets^5,10,15,47– 49^ that include high-resolution geometry typically rely on parametric generation methods, due to the scarcity of real-world building datasets that are both watertight and simulation-ready. However, such datasets are often small in size and limited in the range of design parameters represented.
- **Absence of cross-domain physics simulation data with full spatial coverage**: Most datasets are limited to single-parameter aerodynamic outputs, primarily wind fields. Although this is partly due to computational constraints, it results in the exclusion of other important modalities such as solar radiation, structural response, air temperature, and thermal comfort. A few studies have begun exploring these dimensions concurrently^4,12^, but comprehensive integration remains rare. Furthermore, datasets that provide spatially resolved outputs across ground, surface, and volumetric domains are limited^15,44^, despite such coverage being critical for holistic performance evaluation.

The presented dataset, PRISM, directly addresses these limitations by combining morphological diversity with simulation fidelity. It includes highly diverse building geometries and cross-domain physics simulation outputs, enabling performance-informed modeling during early-stage design. By supporting both geometric variation and validated environmental data, PRISM offers a robust foundation for surrogate modeling, inverse design, and scalable, data-driven analysis in building performance applications.

### PRISM Dataset

**Fig. 1:**
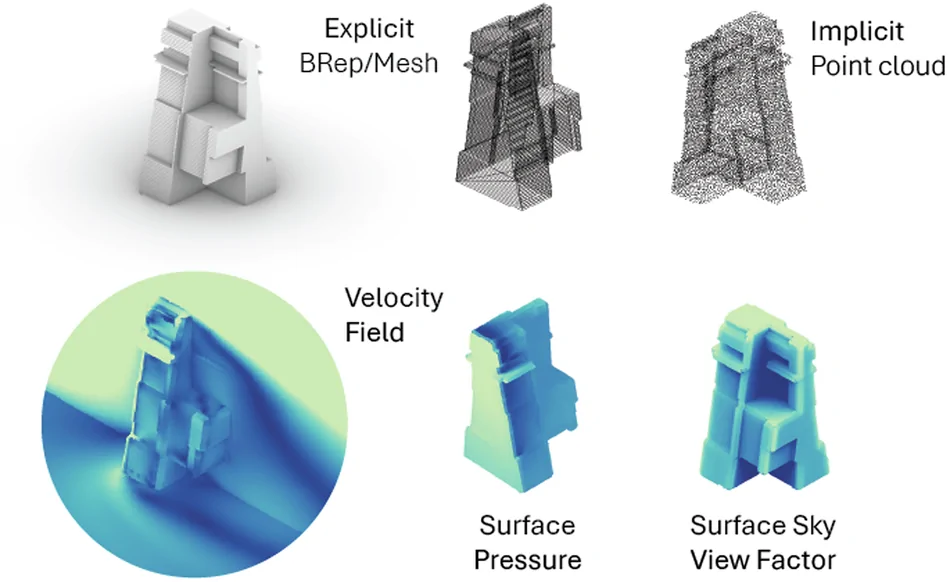
Geometry and performance data modalities for PRISM dataset.

PRISM comprises 4759 procedurally generated building geometries, including 4344 unique forms and 415 matched extrusions, derived from a parametric model with 19 building-level and 8 dataset-level parameters. A representative subset of 831 geometries is paired with high-resolution CFD simulations conducted across 1 to 8 cardinal wind directions per model, with an average of 7.6 directions per case, resulting in 4769 simulation instances. Mesh sizes for the full domain range from 16 to 38 million cells, with an average of 27 million, while the building meshes contain an average of 33000 cells. Sky view factor (SVF) calculations are provided for all 4759 geometries using a sky patch discretization scheme that supports downstream radiation analysis. Each geometry is available in multiple formats, including watertight surface meshes, point clouds, and structured descriptors, and is paired with corresponding velocity, pressure, and SVF-by-patch values, as illustrated in Fig. 1. The dataset totals 5.3 TB in compressed form and exceeds 12.5 TB uncompressed.

## Methods

### Geometry Generation

**Fig. 2:**
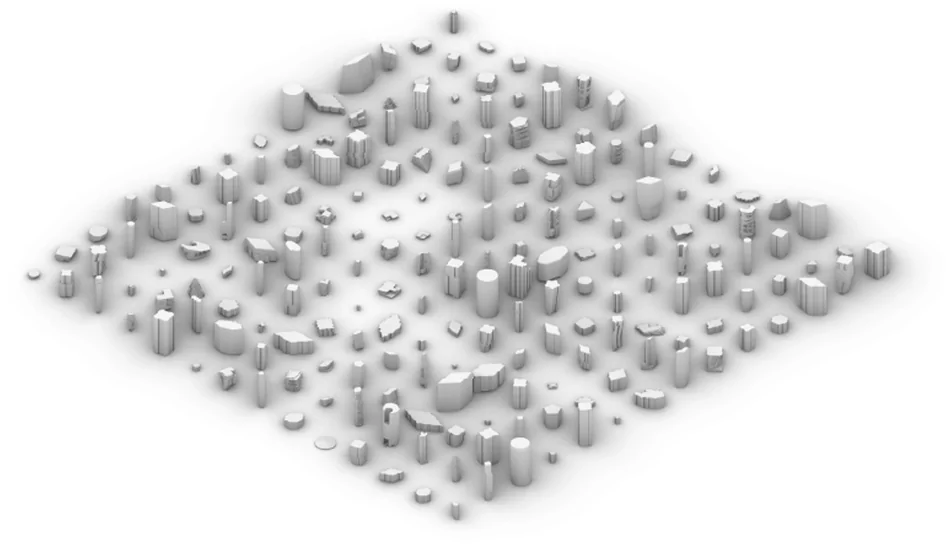
Examples showcasing the range of building morphologies.

A synthetic dataset of building geometries was generated through parametric and procedural modeling using Grasshopper^50^, the visual programming environment working within Rhinoceros 3D^51^, a computer-aided design application. The defined parameters included size (defined by radius ranging from 20 to 60m), height (16 to 300m), proportion (0.5 to 2), shape (defined by polygon sides from 3 to 10 sides plus circular shapes), extrusion type (straight or pointed), corner type (rectangular, chamfered or circular), massing carving (defined by the number, scale, height and building side of the subtractive element(s)), lift-up conditions (no lift-up from the ground up, central/side/opposing cores, arcades, and podium on all site), vertical variation (regular extrusion, pyramid-like (or flipped), smooth graph-based and random variations), horizontal variation (defined by the separation distance from 10 to 50m and the depth of the in-floor variation from 3 to 9m), and roof type (straight, tapered and pointed extrusions). The distribution intensity of parameters was controlled through dataset-level parametrization, enabling a handcrafted approach to ensure morphological diversity representative of building stocks. 5000 geometries were sampled using Latin hypercube sampling from this discrete-continuous design space, yielding 4759 watertight forms, a sample of which is shown in Fig. 2. Simplified extrusion variants were generated for 415 of these.

### Performance Simulations

#### Computational Fluid Dynamics (CFD)

**Fig. 3:**
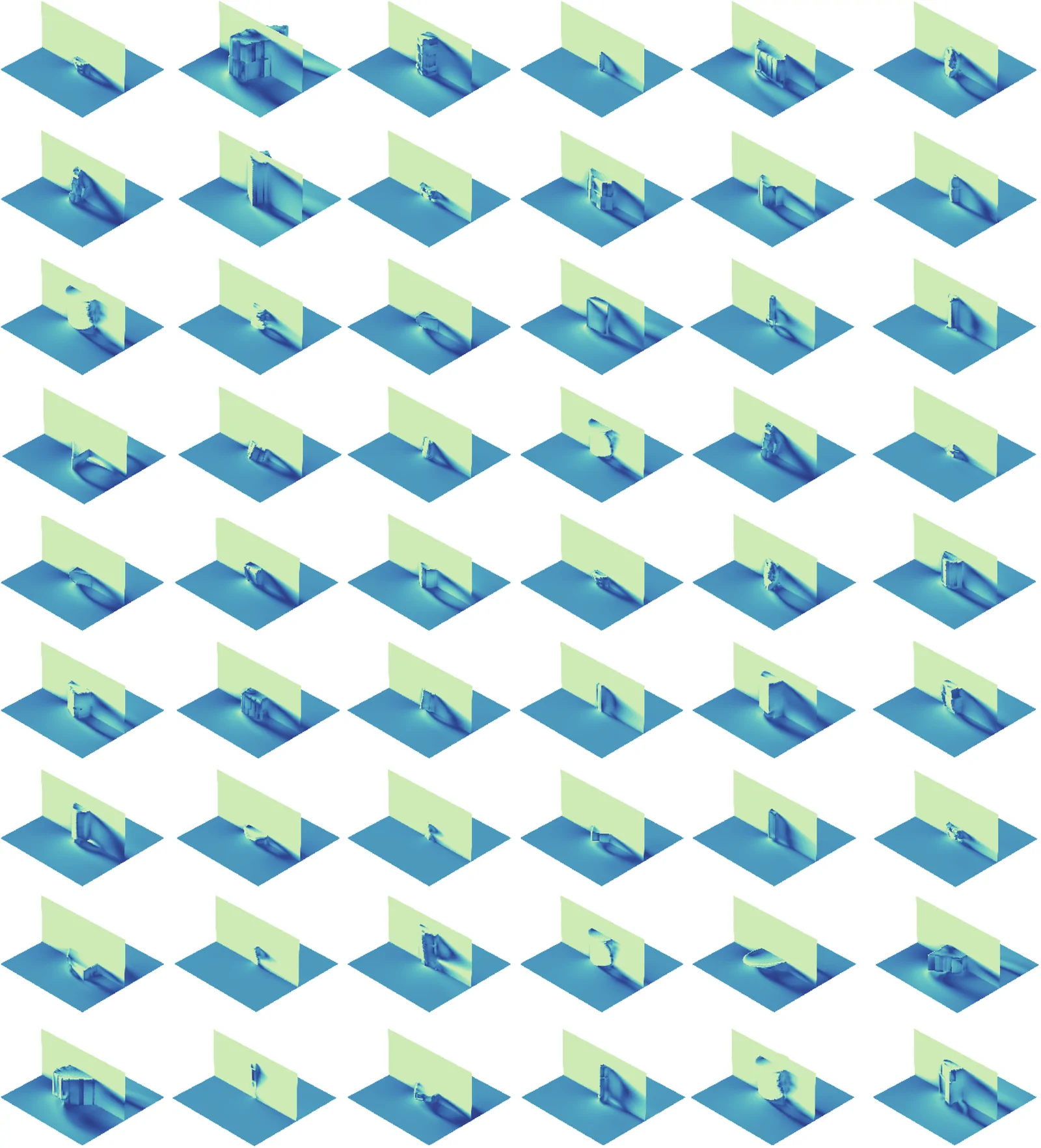
Sample predictions of wind speed fields from the PRISM dataset, illustrating how geometric variations influence flow acceleration and wake structures.

**Fig. 4:**
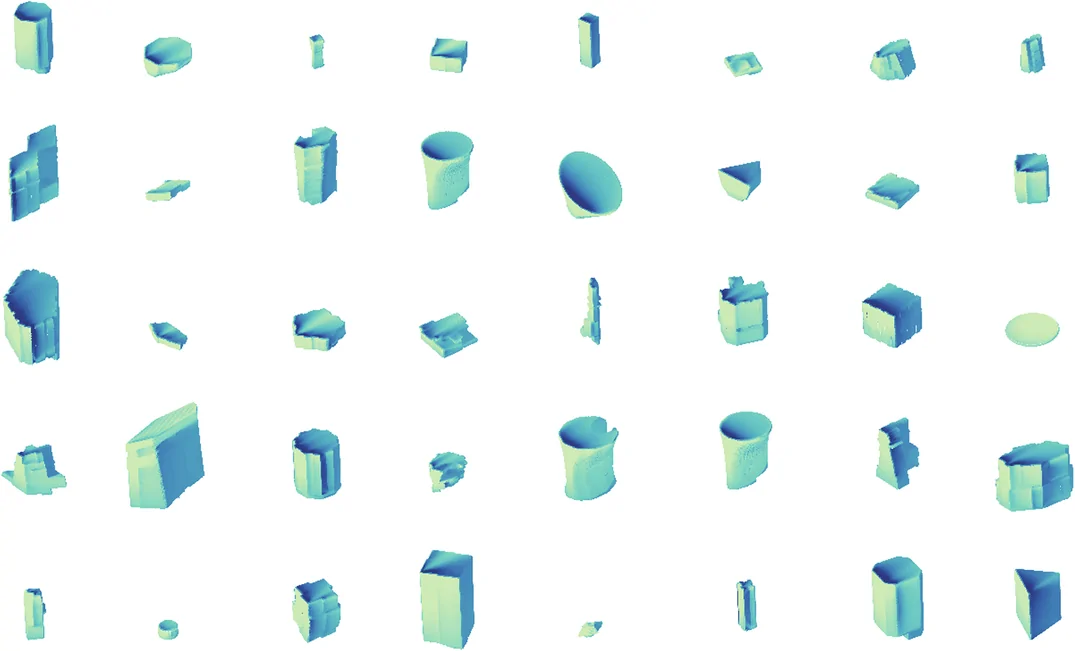
Surface pressure coefficient predictions across multiple building geometries from the PRISM dataset.

CFD simulations were configured according to established guidelines for pedestrian-level wind studies^52,53^, enabling analysis of urban ventilation, comfort, and wind exposure patterns. All CFD simulations in PRISM were conducted using OpenFOAM’s^54^ simpleFoam, a steady-state incompressible RANS solver with the realizable *k*–*ε* turbulence model, following urban flow modeling best practices. Pressure–velocity coupling used the SIMPLE algorithm, with relaxation factors set to 0.7 (pressure) and 0.3 (velocity, *k*, *ε*). Convergence was declared at residuals below 10^−4^ (pressure) and 10^−5^ (velocity, turbulence fields), or after 5000 iterations. Meshing followed a two-stage process: a structured base mesh via blockMesh, then surface-conforming refinement using snappyHexMesh to yield mixed polyhedral grids. The domain was cylindrical, with radius 15 × building height (minimum 500 m) and vertical extent 6 × building height. A base block size of 20 m was used, with four refinement levels in the bounding box, on building surfaces and corners, and on the ground surface within 500 m, and three refinement levels on the remaining ground. Geometry models assumed infinitely thin walls. Across all simulations, an inlet wind speed of 5 m/s at 10 m reference height was used with a terrain roughness of 1.0. Other wind conditions can be estimated using logarithmic wind profile scaling. Simulations assumed neutral, isothermal conditions with no buoyancy or thermal coupling, appropriate for early-stage design and moderate-to-high wind speeds. For each building geometry, simulations were performed for a varying subset of the eight cardinal directions, constrained to an 831-geometry subset of the entire dataset. Simulation setup and mesh generation were automated via the Eddy3D plugin^55^, enabling geometry export and boundary condition specification. Output fields, including wind speed across the flow field and surface pressure coefficients, were exported in .vtk format using foamToVTK for postprocessing. All cases ran on a Linux-based HPC cluster using 32 cores per simulation. A sample set of wind speed fields and surface pressure coefficients for simulated geometries is shown respectively in Figs. 3 and 4, illustrating the influence of geometric features on surrounding wind patterns.

#### Sky View Factor (SVF) via Radiation-Based Sky Discretization Models

**Fig. 5:**
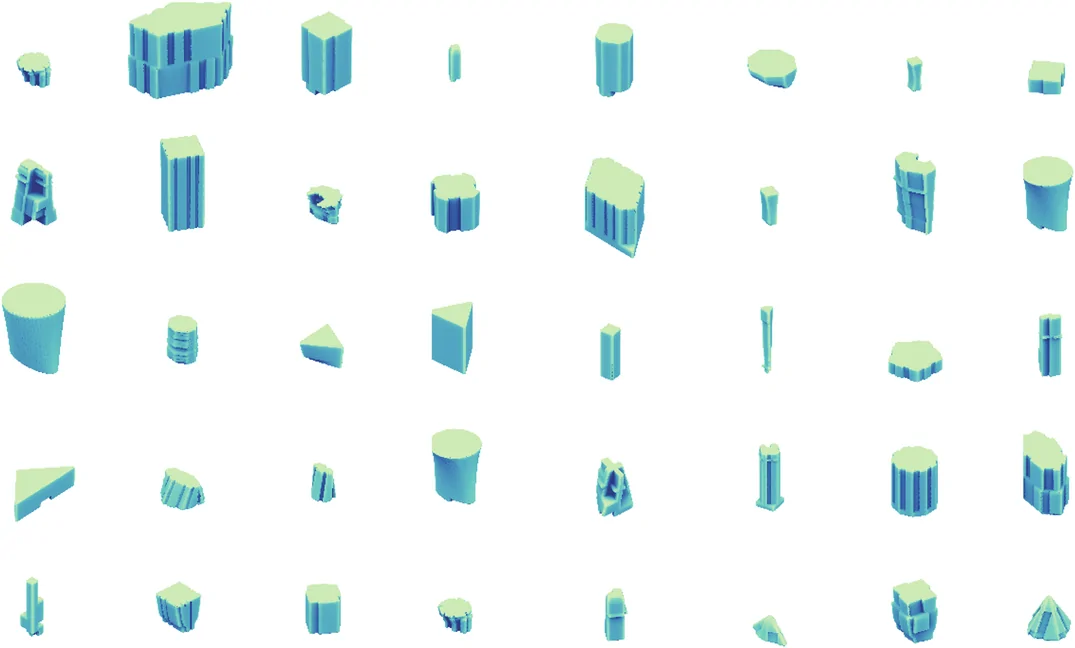
Sky view factor (SVF) distributions for a selection of PRISM building geometries.

The sky view factor (SVF) quantifies the portion of the sky dome visible from a given point, ranging from 0 (fully obstructed) to 1 (completely open). It is commonly used to assess daylight access and evaluate urban microclimate conditions. This metric can be extended to radiation analysis through the Cumulative Sky Matrix (CSM) method^56^, which integrates SVF calculations with sky dome subdivisions defined by radiation-weighted sky models. These subdivisions are coupled with cumulative radiation values derived from climate-based weather data using daylight simulation tools such as Radiance^57^ specifically through its *gendaymtx* function, which generates sky matrices over user-defined periods. This decouples geometry from climate, allowing precomputed SVF values to be reused for radiation estimation across locations and time periods. In this implementation, SVF values are computed via ray-mesh intersection tests between candidate points (sampled on a 2 × 2m horizontal grid offset from building surfaces and ground) and fine sky density of 577 equal patches based on Reinhart sky subdivision scheme^58^. Calculations use trimesh^59^ and pyembree^60^ for spatial queries. Figure 5 shows the cumulative sky view factor distribution for a subset of the dataset.

#### Computational Resources and Cost

The computational cost of generating the performance simulations for this dataset, particularly the high-fidelity CFD cases, was substantial. All simulations were executed on a Linux-based high-performance computing (HPC) cluster using MPI-based parallelization^61^. Each CFD case required between 2 and 12 hours of computation when run in parallel on 32 cores with 128GB of RAM. The total computational effort for simulations, excluding meshing and post-processing, amounted to 860,000 CPU-hours. SVF calculations were performed separately using single-core jobs with 32GB of RAM per core, with the entire SVF component completed in approximately 27 hours.

## Data Records

The dataset includes multi-modal representations of building geometry and performance simulations. Geometries are provided in both explicit and implicit formats: watertight meshes (.obj) and BReps (.3dm); point clouds (including normals) and signed distance fields (SDFs) under various sampling schemes (uniform, near-surface, rejection, and combined schemes) (.npy). Parametric metadata is included in tabular form, along with pre-computed morphology metrics. Performance outputs consist of 3D CFD results (velocity fields in .vtk, surface pressure fields in .vtk), and sky view factors (SVF) per sky patch (.npy, 580-patch Reinhart sky) sampled at both ground and surface levels. The dataset is publicly available under the Creative Commons Attribution 4.0 International (CC BY 4.0) license, permitting use, redistribution, and adaptation for any purpose, including commercial use, provided that appropriate credit is given. It is hosted on the Harvard Dataverse repository^62^ and assigned a persistent DOI to ensure long-term accessibility, citation stability, and reproducibility. It can be accessed through the link: https://doi.org/10.7910/DVN/4IWMVF. The complete dataset is accessed through the Harvard Dataverse landing page, which provides the dataset metadata, licensing information and download links. The data files are stored on the Northeast Storage Exchange (NESE), Harvard Dataverse’s large-data storage infrastructure solution, and are downloaded using Globus, a data transfer service for large research datasets, by following the instructions provided on the dataset page. No data access request or approval is required. To facilitate transparent and structured usage, the dataset is accompanied by metadata using the Croissant metadata schema^63^ and a Datasheet for Datasets^64^, along with a sample geometry instance showcasing all data modalities. Given the scale of the complete dataset archive, practical accessibility and storage requirements may present challenges for some workflows and computational environments. To mitigate this, PRISM is organized into modular subsets to facilitate task-specific use (e.g., surrogate modeling, representation learning) without requiring full download of the 5.3TB archive. Given the dataset’s large scale, a complete sample folder for one building, *building_345*, is provided to facilitate rapid testing and inspection. This sample contains raw simulation outputs (e.g. .vtk files) alongside their pre-processed representations (e.g. .npy arrays), enabling users to quickly test visualization, processing and modeling pipelines directly.

## Technical Validation

To evaluate the sensitivity of discretization choices in simulations on downstream performance metrics, a validation was performed through a mesh sensitivity study for CFD simulations and a sky discretization and sampling study for sky view factor calculations.

### Validation of Simulations

#### CFD Numerical Robustness

To evaluate the sensitivity of the simulation results to mesh resolution, a mesh sensitivity study was conducted using four systematically refined meshes and across 4 geometries with varying degrees of size and geometric complexity. The meshes differ in surface refinement levels (applied to buildings, near-ground, and far-ground surfaces) as well as a refinement box defined as the building bounding box, as summarized in Table 2. The *Very coarse*, *Coarse*, *Medium*, and *Fine* meshes differ by one additional refinement level at each stage, corresponding to a halving of local cell size and an approximate doubling of the number of cells within refined regions. Two additional mesh configurations were investigated, as variations from the *Medium* mesh resolution: *Medium-* doubling the global cell size while maintaining same refinement levels, and *Medium+* adding 4 additional prism layers with the same base cell size and refinement levels. The *Fine* mesh configuration yields an approximate near-surface and near-ground spatial resolution of 0.625 m, with the primary refinement region ranging between approximately 0.625–1.25 m, providing sub-meter near-ground and near-building refinement consistent with commonly adopted urban CFD and pedestrian wind modeling recommendations. It is therefore used as the highest-resolution reference configuration for evaluating relative discretization convergence across mesh resolutions. Table 3 summarizes the mesh parameters, estimated grid sizes, and convergence metrics for velocity and pressure fields. To ensure relevance to downstream performance-informed building application tasks, all reported field quantities are derived from spatially sampled regions around the building of interest. Pedestrian-level wind speed (*U*_*ped*_) is computed as the mean value of velocity magnitudes sampled at 1.5 m, 3m and 4.5m height on a 3  × 3 m grid extending 200 m from the building footprint. Near-surface wind speed (*U*_*s*_) and surface pressure coefficient (*P*_*c*_) are averaged from samples placed on a 2  × 2 m surface-aligned grid with a 0.25 m offset from the building facade. For pressure, the mean of absolute values is used to avoid cancellation between positive and negative contributions. These metrics were selected over more global or volumetric quantities (e.g., drag force) due to their greater interpretability and utility in performance-based applications.

**Table 2:** Mesh refinement configuration for each level in the grid convergence study.

Case	Surface Refinements			Refinement Region
	Building	Ground	Ground Perimeter	Bounding Box
Very coarse	(2 2)	(2 2)	(1 2)	(1 2)
Coarse	(3 3)	(3 3)	(2 3)	(2 3)
Medium	(4 4)	(4 4)	(3 4)	(3 4)
Fine	(5 5)	(5 5)	(4 5)	(4 5)

Surface refinements are reported as OpenFOAM-style level pairs (min max). The refinement region includes the refinement box level and its extent relative to the bounding geometry.

**Table 3:** Grid sensitivity results for wind speed near the building surface (*U*_*s*_), surface pressure coefficient (*P*_*c*_), and pedestrian-level wind speed on the XY grid (*U*_*ped*_).

Case	Cells	*U*_*s*_ [m/s]			*P*_*c*_			*U*_*ped*_ [m/s]			Time/ iter. (s)	Memory (GB)
		Value	Error	% Err.	Value	Error	% Err.	Value	Error	% Err.		
Very coarse	3M	0.39 ± 0.08	0.98 ± 0.14	71	0.29 ± 0.12	0.10 ± 0.02	13	2.61 ± 0.04	1.83 ± 0.09	41	1	8
Coarse	7M	0.41 ± 0.08	0.96 ± 0.12	69	0.30 ± 0.12	0.10 ± 0.02	11	3.02 ± 0.18	1.32 ± 0.08	32	2	15
Medium	20M	0.72 ± 0.08	0.67 ± 0.06	46	0.32 ± 0.12	0.07 ± 0.02	5	3.74 ± 0.10	0.70 ± 0.04	16	7	48
Fine	100M	1.34 ± 0.15	—	0	0.34 ± 0.12	—	0	4.44 ± 0.13	—	0	22	192
Medium+	40M	1.10 ± 0.41	0.73 ± 0.03	18	0.32 ± 0.12	0.07 ± 0.03	5	4.21 ± 0.64	0.54 ± 0.16	5	9	76
Medium-	1M	0.41 ± 0.08	0.96 ± 0.12	69	0.30 ± 0.12	0.10 ± 0.03	11	3.11 ± 0.10	1.33 ± 0.04	30	2	12

Reported values include simulated quantity, relative error and relative percentage error (%) with respect to the *Fine* mesh. These are reported as means and standard deviation across samples and geometries. Computational cost is summarized by time per iteration and memory usage.

Although the finest mesh exhibits the lowest relative errors across all evaluated flow and pressure fields, it entails a substantially higher computational cost, requiring more than three times the memory usage and wall-clock time per iteration compared to the *Medium* resolution mesh. The *Medium* mesh therefore represents a computationally tractable intermediate resolution, offering a large reduction in resource requirements (approximately 75% lower memory usage than the finest mesh) while improving numerical stability and accuracy relative to the coarse configuration. As reported in Table 3, relative errors at the *Medium* resolution are on the order of 16% for pedestrian-level wind speed (*U*_ped_) and 5% for surface pressure coefficients (*P*_*c*_), while remaining comparatively high for near-surface wind speed (*U*_*s*_) at approximately 46%. The introduction of four prism layers reduces the *U*_*s*_ error to approximately 18%, but this refinement is accompanied by a substantial increase in computational cost, including an approximate doubling of total cell count, a 1.5× increase in memory usage, and a 1.3× increase in wall-clock time per iteration, corresponding to an overall cost increase of roughly 2× relative to the *Medium* mesh. Given that pedestrian-level wind speed and surface pressure coefficients constitute the primary quantities of interest for the environmental performance analyses targeted by this dataset, and considering the non-negligible computational overhead associated with further refinement, the *Medium* resolution mesh was adopted as the default configuration. The mesh sensitivity study is provided to transparently report the limitations of this choice and to support downstream users in assessing the resolution’s suitability for their intended applications.

#### SVF/Solar pipelines

This validation assesses the sensitivity of sky view factor (SVF) estimation and derived cumulative solar radiation to sky discretization and patch sampling density.

Tregenza and Reinhart sky model discretizations were evaluated across multiple sampling densities, and errors were quantified for both pedestrian ground-level and building surface regions across ten climatic contexts in Table 4, alongside runtime and memory cost. Results confirm the expected accuracy-cost trade-off: coarser discretizations yield higher SVF and radiation errors, particularly on building surfaces, while finer configurations offer diminishing accuracy gains at substantially increased computational expense. Based on this assessment, the Reinhart sky discretization with one sample per patch is adopted for PRISM. This configuration maintains mean SVF and cumulative radiation errors below 3% and 5% on building surfaces, and below 1% and 0.5% at pedestrian level, while remaining computationally tractable. The selected setup therefore provides sufficient accuracy for downstream performance analysis without incurring unnecessary computational overhead.

**Table 4:** Evaluation across base sky configurations, patch sampling, and target points. Surface (srf) and ground (grd) blocks report sky view factor error (svferr) and radiance error (raderr) as mean ± std (uncertainty shown in smaller font). Runtime (time) and memory (mem) report mean with the maximum in parentheses.

*d*	*p*	*r*	Building Surface				Pedestrian Ground			
			*SVF*_*err*_[%]	*Rad*_*err*_[%]	time (s)	mem (GB)	*SVF*_*err*_[%]	*Rad*_*err*_[%]	time (s)	mem (GB)
T	1	145	5.61 ± 21.76	22.76 ± 155.21	5 (9)	0.2 (0.3)	0.72 ± 1.08	1.60 ± 7.49	15	0.6
**R**	**1**	**577**	**2.55 ± 14.73**	**5.05 ± 65.12**	**7(14)**	**0.7(1.3)**	**0.23 ± 0.32**	**0.32 ± 1.35**	**18**	**2.3**
R	2	1154	1.72 ± 9.93	3.44 ± 31.25	9 (19)	1.4 (2.6)	0.14 ± 0.20	0.20 ± 0.74	24	4.6
R	4	2308	1.17 ± 7.80	2.05 ± 18.41	15 (32)	2.8 (5.2)	0.08 ± 0.12	0.11 ± 0.42	36	9.2
R	8	4616	0.81 ± 5.54	3.39 ± 74.72	27 (56)	5.6 (10.4)	0.05 ± 0.08	0.07 ± 0.28	61	18.3
R	16	9232	0.00 ± 0.00	0.00 ± 0.00	49 (103)	11.1 (20.8)	0.00 ± 0.00	0.00 ± 0.00	239	36.6

*Notes:* For errors, the small-font value is the standard deviation; for time/memory it is the maximum observed

### Morphological Characterization

To assess morphological diversity and shape complexity, a set of 3D shape metrics defined for the built environment^65^ were computed for PRISM and compared to two public datasets: the Boston 3D City Model^28^ and the Netherlands 3D BAG dataset^29^. Pre-computed values were used for the Netherlands dataset (183,500 LOD2.2+ buildings)^65,66^, while metrics were calculated for 14,618 LOD2+ Boston buildings and 4,022 from PRISM.

These metrics capture properties such as roughness, compactness, and spatial distribution. Higher values indicate either increased geometric complexity (e.g., fractality, roughness, exchange, spin, depth, dispersion) or greater regularity (e.g., hemisphericality, convexity, cuboidness, proximity, girth).

**Fig. 6:**
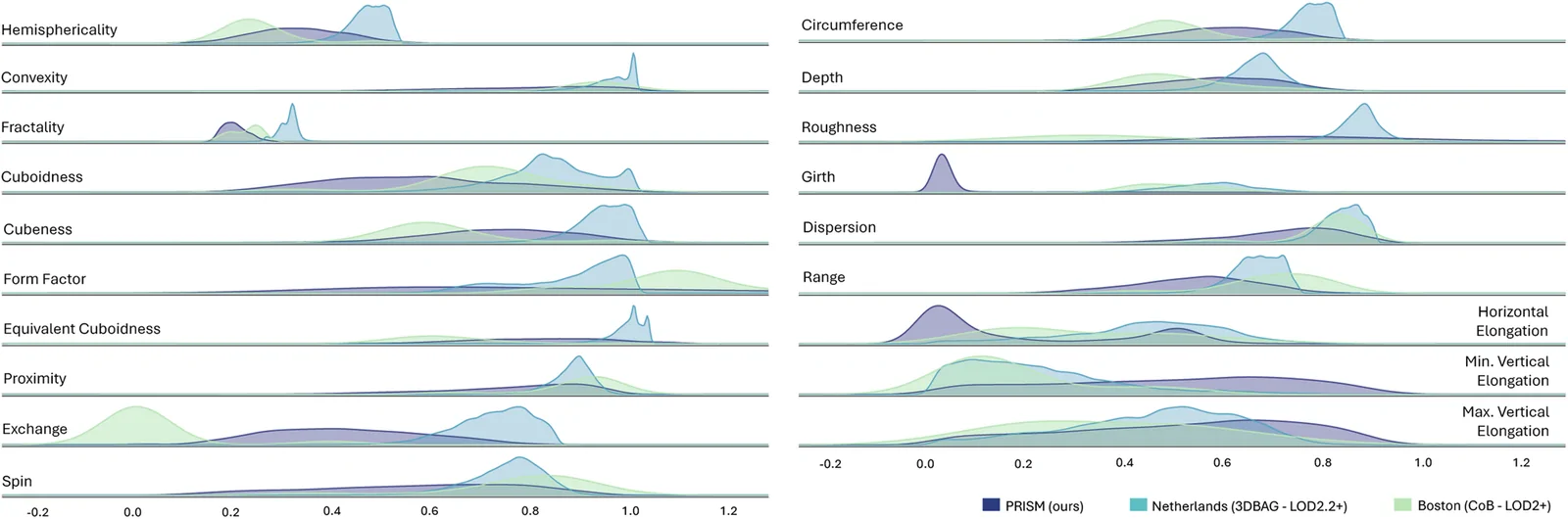
Distribution of 3D shape complexity metrics for the PRISM, Netherlands (LOD2.2+), and Boston (LOD2+) datasets. PRISM shows broader distributions and higher values for metrics capturing deviation from ideal forms and volumetric articulation, indicating greater morphological diversity. It also differs notably in girth, suggesting more recessed and protruded geometry than typical city-scale models.

Figure 6 shows the distribution of 3D shape complexity metrics across the three datasets, revealing substantial and distinctive variability that reflects differences in modeling conventions, urban typologies, and levels of geometric abstraction. The PRISM dataset displays broader distributional coverage across most metrics, particularly those capturing deviation from idealized forms, such as hemisphericality, cuboidness, and convexity, indicating a greater diversity in volumetric configurations. Metrics associated with surface articulation and detail, such as fractality and roughness, are generally higher in the Netherlands dataset, which is expected given its geospatial origin; such models tend to retain fine-grained facade irregularities, unlike parametric models which often produce smoother surfaces. PRISM also stands out in its girth values, which reflect the presence of deep recesses and protrusions, features that appear largely absent from the Boston and Netherlands datasets. This may be due to the fact that, at LOD2+, city models tend to follow simplified geometric abstractions that do not always represent sub-building articulation. As such, the observed differences likely reflect not only variation in underlying building forms but also differences in how geometric detail is represented across modeling pipelines.

**Fig. 7:**
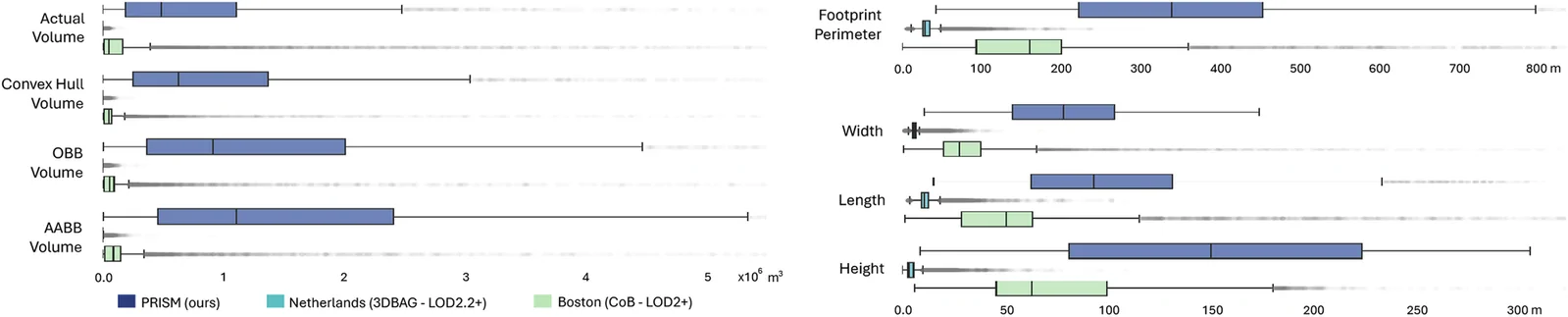
Distribution of basic shape metrics (volume, width, length, perimeter) across the PRISM, Netherlands, and Boston datasets. PRISM shows broader variation, reflecting a wider range of building typologies and scales.

Basic shape descriptors,such as volume, width, length, and perimeter, are compared across the three datasets in Fig. 7. The PRISM dataset exhibits a significantly wider distribution across these metrics, reflecting its design objective to capture a broad range of urban building typologies. Unlike the Netherlands dataset, which is heavily skewed toward smaller, likely single-story buildings, PRISM includes structures that vary widely in scale, height, and footprint, supporting more diverse and transferable modeling applications beyond a single-city context.

### Performance Characterization and Intra-Dataset Variation

Capturing variation in morphological characteristics alone is insufficient for performance-driven applications, it is equally important that the dataset reflects diversity in physical behavior across building instances. Since building performance tasks rarely depend on single summary metrics, understanding the spatial distribution of performance variables, such as wind speed, radiation, or sky view factor, across surfaces and pedestrian areas is critical. This enables identification of phenomena such as buildings contributing to large zones of high wind speeds, localized stagnation areas, floor-specific ventilation potential, or excessive solar exposure.

**Fig. 8:**
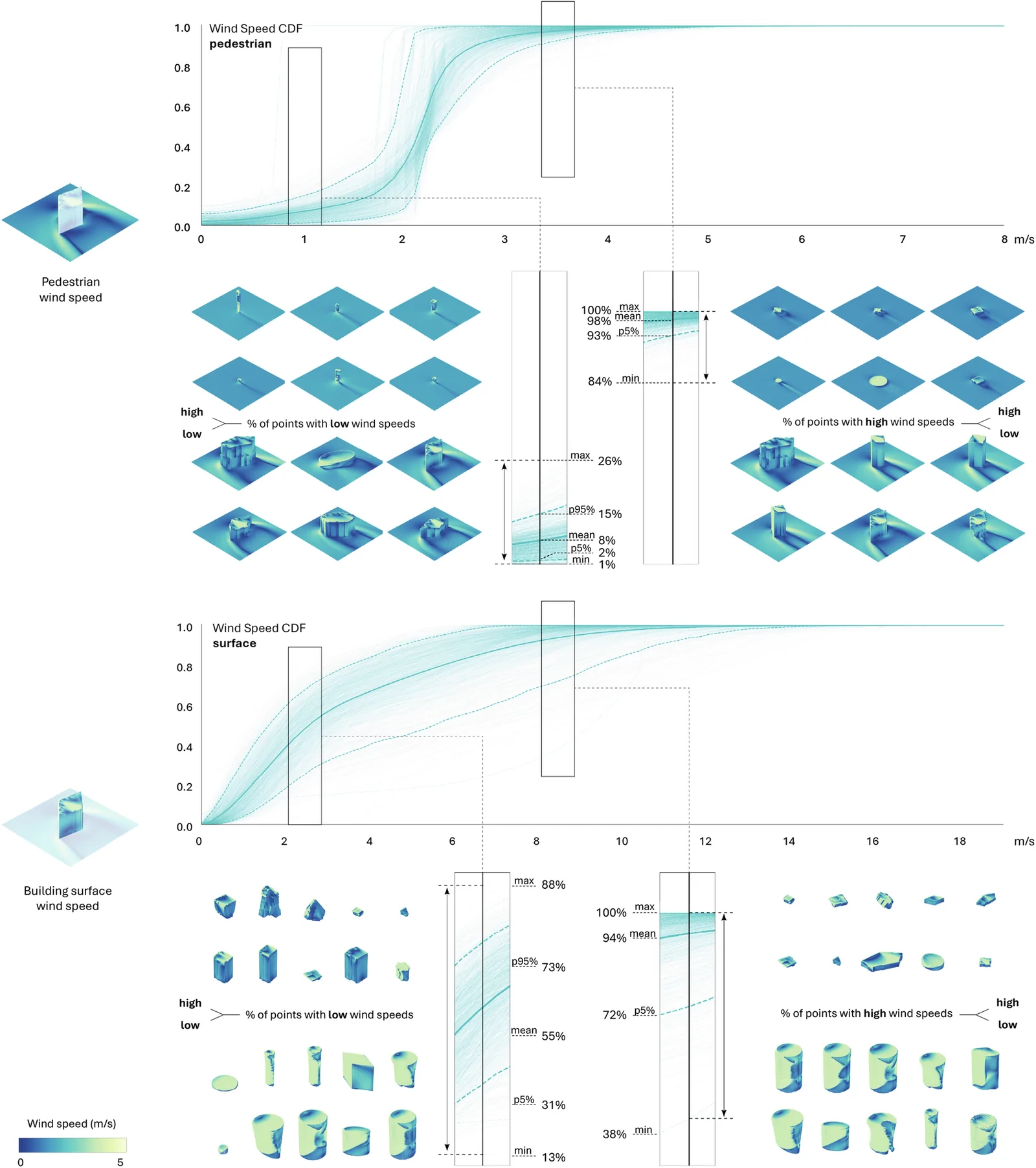
Cumulative distribution of wind speed values at pedestrian-level and near-surface points across the dataset. The range reflects diverse aerodynamic responses driven by variation in form and exposure.

**Fig. 9:**
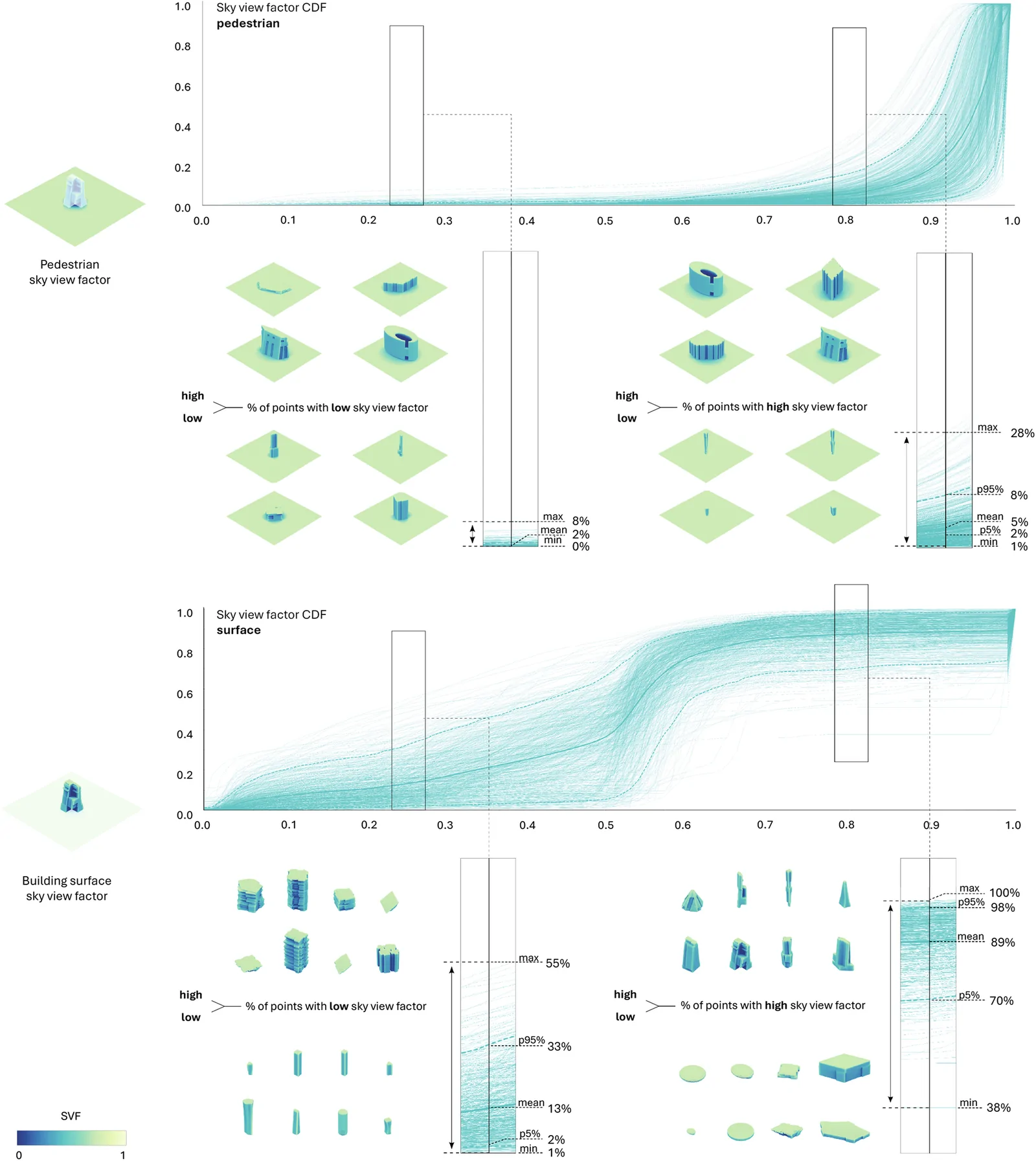
Cumulative distribution of sky view factor values at pedestrian-level and near-surface points across the dataset. The results highlight the dataset’s inclusion of both compact and articulated geometries relevant for evaluating solar and radiative conditions.

To evaluate performance variability, cumulative distribution functions (CDFs) were computed for key environmental metrics across the dataset. Figures. 8 and 9 show CDFs for wind speed and sky view factor at pedestrian- and surface-level points, respectively. Representative building instances are visualized at selected percentiles to illustrate the range of spatial outcomes. The dataset enables identification of recurring form-performance relationships, such as elevated wind exposure near circular forms or reduced sky view factors in highly articulated geometries, and supports more nuanced, cross-parametric analysis of shape and performance across a broad morphological spectrum.

### Learning-Based Applications

To evaluate the suitability of PRISM for data-driven surrogate modeling, two deep learning architectures are benchmarked on the dataset: Transolver^67,68^, a transformer-based model for geometry-aware field inference, and GraphSAGE^69^, a graph neural network that incorporates edge-based message passing over spatial neighborhoods. Both architectures have shown success in other physics-informed learning domains but are not yet widely applied to building-scale CFD tasks. The models operate on unstructured mesh points annotated with coordinates, signed distance values, and surface normals. In GraphSAGE, edge connectivity encodes local geometric relationships between neighboring mesh points. To manage the memory constraints of PRISM’s large meshes (16–38 million cells per geometry), a uniformly preprocessed subset of 1 million points was used for training. This subset retains all surface points, excludes distant regions beyond 550 m, and applies a distance-weighted sampling strategy. At each training step, a random subsample was drawn: 520,000 points per batch for Transolver and 800,000 for GraphSAGE. Models were trained for 150 epochs using the Adam optimizer (lr = 10^−3^, batch size = 1) and evaluated on 402 held-out test instances. Following the setup in^68^, both models used 4 attention layers, 8 heads, 256 hidden channels, and 32 spatial slices. The training objective included weighted terms for velocity, surface pressure, and velocity magnitude following 10:10:1 ratio. Training was performed on a subset of 3,535 samples and evaluated on a held-out test set of 350, with the task of predicting velocity and surface pressure fields (*U* and *P*). To assess model generalization beyond the training distribution, performance was also measured on a real-world out-of-distribution (OOD) dataset comprising unseen building geometries and independently simulated CFD fields. The OOD dataset included 10 buildings drawn from two public sources: 6 from the Boston 3D Buildings dataset^28^ and 4 from a 1 km^2^ sample of London provided by AccuCities^70^. Candidate geometries were filtered for watertightness, LoD3+ complexity, and morphological diversity. High-resolution CFD simulations were performed for each geometry following the same preprocessing, meshing, and solver configuration used in PRISM. While limited in scale and not intended to comprehensively represent the diversity and contextual complexity of real urban environments, this OOD set provides an initial benchmark for evaluating whether geometry-performance relationships learned from PRISM exhibit transferability to previously unseen real-world morphologies. In this evaluation, *t*_*g*_, *t*_*c*_, and *t*_*r*_ denote the validation, test, and real sets, respectively. The validation and test sets are drawn from PRISM, which are based on a fixed train/validation/test partition used throughout the experiments. The *t*_*g*_ validation set was employed exclusively for model monitoring and selection during training and *t*_*c*_ correspond to held-out synthetic test data, while *t*_*r*_ provides an OOD benchmark to assess relevance for real-world learning contexts. The focus here is not on achieving state-of-the-art accuracy, as these models were not hyperparameter-tuned or exhaustively evaluated, but on demonstrating PRISM’s potential for surrogate modeling, its ability to yield reasonable prediction errors, and its usability for large-scale applications.

Table 5 summarizes mean absolute error (MAE) and the 50th percentile of the absolute percentage error (*p*_50_-APE) for field wind speed predictions (*U*_*f*_) and surface pressure coefficients (*P*_*s*_) for the Transolver and GraphSAGE models. The results show that percentage errors for field wind speeds and surface pressure coefficients can be as low as 3% and 20%, respectively, translating to 0.15-0.25 m/s for *U* and 0.10-0.15 for *P*. Performance trends remain consistent across the three test sets, suggesting that models trained on PRISM can capture geometry-performance relationships that exhibit some degree of transferability to previously unseen real-world morphologies. In some cases, the synthetic test set exhibits larger errors, which can be attributed to the higher geometrical complexity of the synthetic dataset compared to the real-world samples. Given the limited scale of the presented OOD evaluation, however, these results should be interpreted as an initial indication of transfer potential rather than a comprehensive assessment of real-world generalization.

**Table 5:** PRISM for data-driven surrogate modeling: predictive performance of physics fields (velocity and surface pressure prediction) across three test sets *t*_*g*_ (synthetic-validation), *t*_*c*_ (synthetic-test), and *t*_*r*_ (real), described in terms of mean absolute error (MAE) [m/s for *U* and unitless for *P*] and the 50th percentile of the absolute percentage error (*p*_50_-APE).

Model	Params	Metric	*t*_*g*_		*t*_*c*_		*t*_*r*_	
			*P*_*s*_	*U*_*f*_	*P*_*s*_	*U*_*f*_	*P*_*s*_	*U*_*f*_
Transolver	2,000,548	*p*_50_-APE	0.1851 ± 0.128	0.0292 ± 0.004	0.1807 ± 0.110	0.0356 ± 0.011	0.3190 ± 0.173	0.0291 ± 0.013
		MAE	0.0907 ± 0.027	0.1914 ± 0.055	0.0962 ± 0.032	0.2456 ± 0.094	0.0972 ± 0.009	0.1585 ± 0.035
GraphSAGE	929,284	*p*_50_-APE	0.2462 ± 0.221	0.0202 ± 0.008	0.2099 ± 0.115	0.0241 ± 0.013	0.3169 ± 0.155	0.0289 ± 0.019
		MAE	0.1176 ± 0.042	0.1899 ± 0.060	0.1187 ± 0.034	0.2496 ± 0.125	0.1206 ± 0.021	0.1689 ± 0.034

Results show predictive capabilities and demonstrate out-of-distribution potential.

## Usage Notes

### Utility: Performance Metrics and Applications

Each building instance in the dataset is associated with three primary simulation outputs: the velocity field ${\bf{U}}\in {{\mathbb{R}}}^{(b,n,3)}$, scalar pressure field $P\in {{\mathbb{R}}}^{(b,n,1)}$, and sky view factor by sky patch $\,{\rm{SVF}}\,\in {{\mathbb{R}}}^{(b,n,580)}$, where *b* denotes the number of buildings and *n* the number of points. These outputs are derived from the simulation setup described in the *Methods* section and technically validated in *Validation of Simulations* section, and serve as a shared representation across multiple performance-related tasks. While simulation requirements vary across domains, ranging from preferred turbulence models and wall treatment schemes to resolution and coupling with thermal or radiative processes, this dataset is intended to support a wide range of early-stage design applications.

**Fig. 10:**
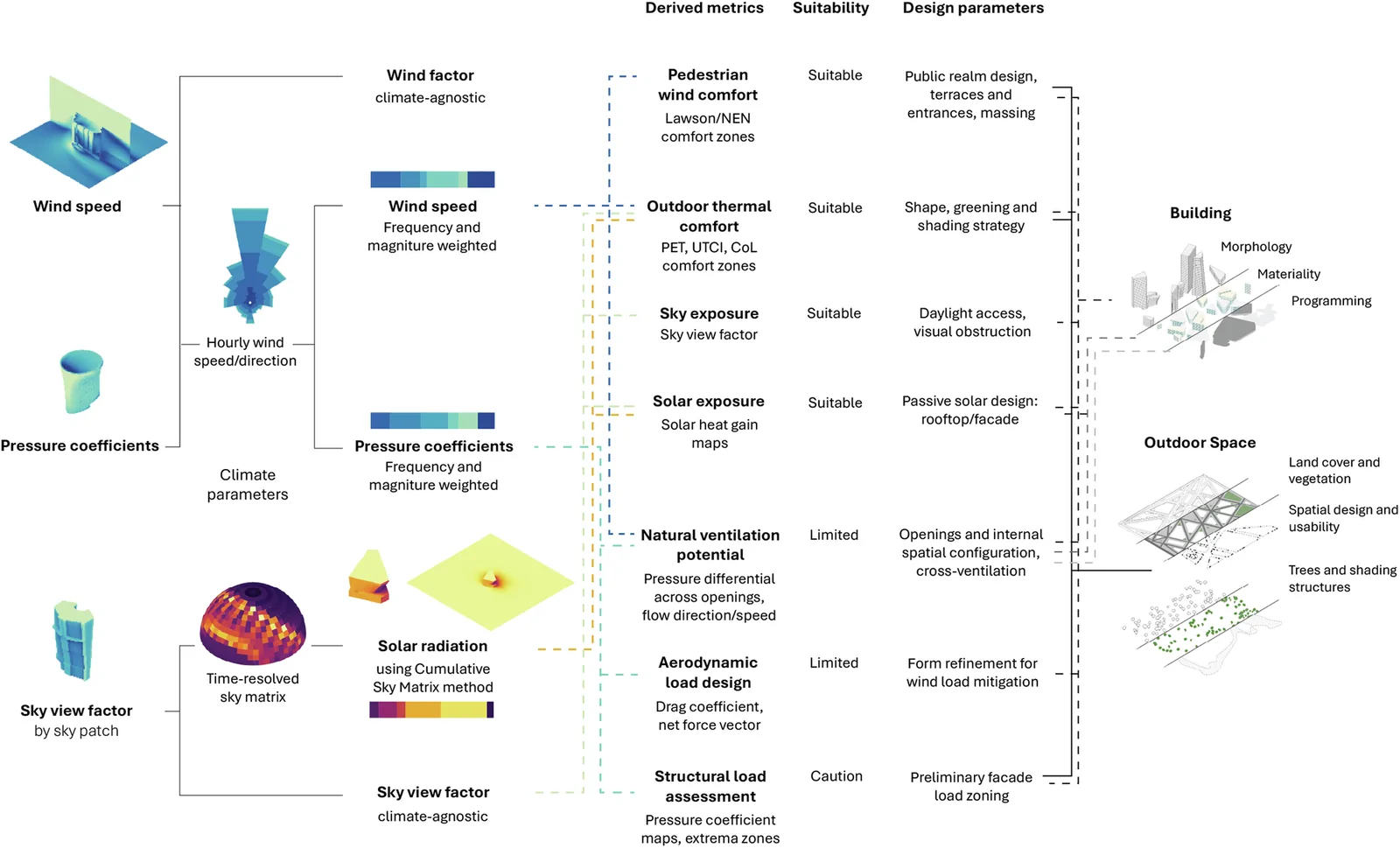
PRSIM use cases: performance-based applications, suitability and limitations.

Figure 10 illustrates how the raw fields can be used directly (climate-agnostic) or composed with climate inputs to derive application metrics. Examples include wind-speed magnitude, exceedance probabilities and comfort classes (from directional distributions and log-profile scaling), and patch-weighted irradiance or daylight proxies via SVF aggregation over the 577-patch Reinhart sky. Table 6 details limitations induced by the numerical choices (e.g., steady-state RANS, wall functions, discretization) to guide appropriate use in early-stage design.

**Table 6:** Simulation suitability and utility of the performance dataset across building performance application domains.

Derived Metrics	Suitability	Limitations	Design Phase
**Pedestrian wind comfort** (Lawson/NEN comfort zones)	Suitable	**Transient behavior**: Steady-state RANS does not capture gusts, fluctuations and unsteady separation.	Public realm design, terraces and entrances, massing.
**Outdoor thermal comfort** (PET, UTCI, CoL comfort zones)	Suitable	**Thermal decoupling**: Wind and thermal effects are modeled independently, convective interactions not captured.	Shape, greening and shading strategy
**Sky exposure** (Sky view factor)	Suitable	**Sky discretization**: Assumes fixed sky patch visibility, view within patches is not directionally resolved.	Daylight access, visual obstruction
**Solar exposure** (Seasonal/annual solar gain maps)	Suitable	**Materiality and dynamic shading**: Time-averaged conditions, dynamic shading and material reflectance not captured	Passive solar design: rooftop/facade
**Natural ventilation potential** (Pressure differentials across openings, flow direction/speed)	Limited	**Near-surface resolution limitations**: pressure differentials sensitive to wall treatment and turbulence model assumptions, not tailored for NV.	Openings and internal spatial configuration, cross-ventilation
**Aerodynamic load design** (Drag coefficient, net force vector)	Limited	**Transient behavior**: Time-averaged simulations cannot resolve vortex shredding, peak forces or dynamic pressure fluctuations	Form refinement for wind load mitigation
**Structural load assessment** (Pressure coefficient maps, extrema zones)	Caution	**Local near-surface details**: wall functions and mesh resolution can underpredict peaks at edges, corners and suction zones.	Preliminary façade load zoning

The table highlights typical design-phase use cases supported by the dataset, associated analysis capabilities, and limitations stemming from numerical modeling choices.

### Limitations

The dataset provides a large-scale corpus of synthetically generated building geometries with high morphological diversity and their corresponding performance fields; however, several limitations remain. First, the number of unique samples is constrained by the substantial computational cost of high-fidelity simulation. The simulation configuration, including boundary conditions and turbulence model, should be evaluated for compatibility with specific downstream tasks. While the dataset is designed to explore the influence of geometric complexity on environmental performance, it does not attempt to match the statistical distribution of real-world buildings or typologies. As such, the use of purely synthetic geometries for tasks like surrogate modeling may limit generalization, and domain-specific validation is necessary to assess predictive reliability. The presented OOD evaluation is therefore intended as an initial indication of transfer potential rather than a comprehensive validation of real-world deployment readiness. This dataset is intended as an enabling resource for geometry-aware learning and early-stage design exploration, rather than as a substitute for project-specific, high-fidelity modeling.

## Data Availability

The PRSIM dataset is hosted on the Harvard Dataverse repository^62^ and can be accessed currently through the link: https://doi.org/10.7910/DVN/4IWMVF. The Harvard Dataverse landing page provides the dataset metadata, licensing information and download links. The data files are stored on the Northeast Storage Exchange (NESE), Harvard Dataverse's large-data storage infrastructure solution, and are downloaded using Globus, a data transfer service for large research datasets, by following the instructions provided on the dataset page. No data access request or approval is required. The dataset is publicly available under the Creative Commons Attribution 4.0 International (CC BY 4.0) license, permitting use, redistribution, and adaptation for any purpose, including commercial use, provided that appropriate credit is given.

## Code availability

Supporting code for processing, exploration, and testing of the PRISM dataset is provided to facilitate its usage and inspection. The code repository is currently accessible through the link: https://github.com/samokhtar/prism. It is released under the MIT License, which permits free use, modification, and distribution of the software, provided that the copyright and license notice are included in all copies. The repository contains tools for processing and visualization, morphology characterization, dataloader setups for direct integration with deep learning frameworks, and baseline implementations for learning-based surrogate modeling.
